# Enhancement of gene expression noise from transcription factor binding to genomic decoy sites

**DOI:** 10.1038/s41598-020-65750-2

**Published:** 2020-06-04

**Authors:** Supravat Dey, Mohammad Soltani, Abhyudai Singh

**Affiliations:** 1 Department of Electrical and Computer Engineering, University of Delaware, Newark, DE 19716 USA; 2 Department of Biomedical Engineering, University of Delaware, Newark, DE 19716 USA; 3 Department of Mathematical Sciences, University of Delaware, Newark, DE 19716 USA; 4 Center for Bioinformatics and Computational Biology, University of Delaware, Newark, DE 19716 USA

**Keywords:** Systems biology, Cellular noise, Stochastic modelling, Gene expression, Gene regulation

## Abstract

The genome contains several high-affinity non-functional binding sites for transcription factors (TFs) creating a hidden and unexplored layer of gene regulation. We investigate the role of such “decoy sites” in controlling noise (random fluctuations) in the level of a TF that is synthesized in stochastic bursts. Prior studies have assumed that decoy-bound TFs are protected from degradation, and in this case decoys function to buffer noise. Relaxing this assumption to consider arbitrary degradation rates for both bound/unbound TF states, we find rich noise behaviors. For low-affinity decoys, noise in the level of unbound TF always monotonically decreases to the Poisson limit with increasing decoy numbers. In contrast, for high-affinity decoys, noise levels first increase with increasing decoy numbers, before decreasing back to the Poisson limit. Interestingly, while protection of bound TFs from degradation slows the time-scale of fluctuations in the unbound TF levels, the decay of bound TFs leads to faster fluctuations and smaller noise propagation to downstream target proteins. In summary, our analysis reveals stochastic dynamics emerging from nonspecific binding of TFs and highlights the dual role of decoys as attenuators or amplifiers of gene expression noise depending on their binding affinity and stability of the bound TF.

## Introduction

The level of a gene product can show remarkable cell-to-cell differences within an isogenic cell population exposed to the same external environment^1–9^. This intercellular variation has been referred to as gene expression noise and is attributed to biochemical processes operating with low-copy number components, such as the number of the promoter, mRNA, and protein for a given gene. The noise in expression critically impacts the functioning of diverse cellular processes in both beneficial and detrimental ways. For example, on one hand the noise is detrimental for developing embryos^7,10,11^, organismal fitness^12^ and has been connected to disease phenotypes^13,14^. On the other hand, noisy expression drives phenotypic heterogeneity between otherwise genetically-identical cells, allowing them to hedge their bets against environmental uncertainties^5,8,15–24^. While there has been considerable work uncovering the role of transcription/translation processes together with molecular feedbacks in driving expression noise^25–28^, how nonspecific binding of a protein shapes this noise remains poorly understood.

A fundamental step in gene regulation is the binding of a transcription factor (TF) to its target promoter^29–31^. Besides this specific binding, TFs also bind to other non-functional high-affinity sites spread across the genome. These spurious binding sites, known as transcription factor decoys, can be present in different abundances with various binding affinities^32–35^. The regularity role of decoys through molecular sequestration of TFs has been experimentally demonstrated via synthetic circuits in *Escherichia coli*^36^ and *Saccharomyces cerevisiae*^37^, and not surprisingly, the TF-inhibiting activity of decoys has tremendous therapeutic potential^38–41^. An immediate consequence of TF sequestration by decoys is the modulation of TF’s stability. Depending on the context, decoy binding can either enhance or reduce the stability of the TFs^42^. For certain TFs, such as MyoD, the DNA binding provide increased stability against degradation^43,44^. On the other hand, for VP16 in *Saccharomyces cerevisiae*, the bound TFs become more prone to degradation by the ubiquitin-mediated proteolysis^45^. A key focus of this work is to investigate how the relative stability of bound vs. unbound TF affects both the extent and time-scale of fluctuations in the level of a given TF.

Prior theoretical work on this topic has highlighted the role of decoys as noise buffers, in the sense that, the presence of decoys attenuates random fluctuations in number of freely (unbound) available copies of the TF^42,46–55^. However, these results are based on the assumption that sequestration of TF at a decoy site protects the TF from degradation. Relaxing this assumption to consider an arbitrary decay rate of the bound TF, we uncover a novel role of decoys as both noise amplifiers and buffers. We systematically characterize the parameter regimes leading to these contrasting roles in terms of the bound vs. free-TF stability, number and affinity of decoy sites. Finally, we study noise transmission from the TF to a downstream target protein reporting counterintuitive effects in some cases, for example, decoys amplify noise in the level of a TF but reduces noise in the level of the TF’s downstream target protein.

## Results

### Model formulation

**Figure 1:**
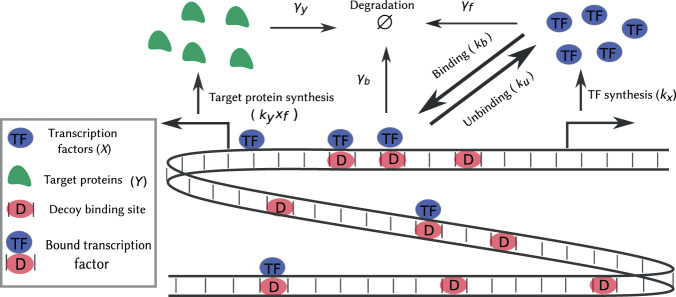
Model schematic for investigating the impact of nonspecific transcription factor binding on expression noise. A genome with several decoy binding sites where transcription factors (TFs) bind reversibly, is depicted. The synthesis of TFs occurs in stochastic bursts. Both the free and bound TFs are subject to degradation. The free TFs activate a target gene and regulate its bursty protein synthesis.

To study the role of decoy binding sites, we consider a TF that is synthesized in stochastic bursts (Fig. 1). Such bursty expression of gene products has been experimentally observed in diverse cell types^56–63^, and corresponds to distinct mechanisms at the transcriptional and translation level. For example, a promoter randomly switches from a transcriptionally inactive to an active state, and quickly turns off after producing a bursts of mRNAs^59,64–66^. Moreover, assuming short-lived mRNAs, each synthesized mRNA is rapidly degraded after translating a burst of proteins^67–69^. We phenomenologically model the combined effect of transcriptional and translational bursts by considering a Poisson arrival of burst events with rate *k*_*x*_, and each event creates *B*_*x*_ molecules, where *B*_*x*_ is an independent and identically distributed non-negative random variable following an arbitrary distribution^70–75^. More specifically, *B*_*x*_ = *i* with probability *α*_*x*_(*i*) where *i* ∈ {0, 1, 2, …}. We consider a general form for the burst size distribution *α*_*x*_(*i*) throughout the paper, except for plotting and simulation purposes where *α*_*x*_(*i*) follows a geometrical distribution^69,76–78^.

Consider *N* decoy binding sites in the genome, with the TF binding/unbinding to each decoy site with rates are *k*_*b*_ and *k*_*u*_, respectively. As motivated earlier, we allow for both the free (unbound) and bound TF to decay with rates *γ*_*f*_ and *γ*_*b*_, respectively. Let *x*_*f*_(*t*) denote the number of free TF molecules, and *x*_*b*_(*t*) the number of bound TFs at time *t* inside a single cell. Then, the stochastic evolution of *x*_*f*_(*t*) and *x*_*b*_(*t*) is governed by the following probabilities

1a $${\rm{TF}}\,{\rm{synthesis}}:{\rm{Probability}}\{{x}_{f}(t+dt)={x}_{f}(t)+i\}={k}_{x}{\alpha }_{x}(i)dt,$$

1b $$\begin{array}{c}{\rm{TF}}\,{\rm{binding}}:{\rm{Probability}}\{{x}_{f}(t+dt)={x}_{f}(t)-1,\,{x}_{b}(t+dt)={x}_{b}(t)+1\}\\ \,={k}_{b}{x}_{f}(t)(N-{x}_{b}(t))dt,\end{array}$$

1c $${\rm{TF}}\,{\rm{unbinding}}:{\rm{Probability}}\{{x}_{f}(t+dt)={x}_{f}(t)+1,\,{x}_{b}(t+dt)={x}_{b}(t)-1\}={k}_{u}{x}_{b}(t)dt,$$

1d $${\rm{Free}}\,{\rm{TF}}\,{\rm{degradation}}:{\rm{Probability}}\{{x}_{f}(t+dt)={x}_{f}(t)-1\}={\gamma }_{f}{x}_{f}(t)dt,$$

1e $${\rm{Bound}}\,{\rm{TF}}\,{\rm{degradation}}:{\rm{Probability}}\{{x}_{b}(t+dt)={x}_{b}(t)-1\}={\gamma }_{b}{x}_{b}(t)dt.$$

Each equation here defines a stochastic event that occurs with a certain probability in the small time interval (*t*, *t* + *dt*], and whenever the event occurs, the population counts change by discrete integer amounts. Based on the underlying stochastic chemical kinetics, these occurrence probabilities are either independent (as in 1a), or linearly/nonlinearly dependent on the molecular counts. For readers convenience, we provide a list of model parameters along with their description in Table 1. Expressing *γ*_*b*_ in terms of *γ*_*f*_ as *γ*_*b*_ = *βγ*_*f*_, *β* = 0 corresponds to no decay of bound TF, and *β* = 1 corresponds to equal degradation rates for both the free and bound TF. The key focus of this work is to understand the stochastic dynamics of *x*_*f*_(*t*) in different regimes of *β*.

**Table 1:** Summary of notation used.

Parameter	Description	Parameter	Description
*x*_*f*_	Free TF count	*x*_*b*_	Bound TF count
*N*	Total decoy binding sites	*k*_*x*_	TF burst frequency
*k*_*b*_	TF binding rate	*k*_*u*_	TF unbinding rate
*γ*_*b*_	Bound TF degradation rate	*γ*_*f*_	Free TF degradation rate
*β*	*γ*_*b*_/*γ*_*f*_	*k*_*d*_	Dissociation constant (*k*_*u*_/*k*_*b*_)
*y*	Target protein count	*k*_*y*_*x*_*f*_	Target protein burst frequency
*γ*_*y*_	Target protein degradation rate	*α*_*x*_ (*α*_*y*_)	Burst size distribution of *X*(*Y*)
$\langle {B}_{x}\rangle \,(\langle {B}_{y}\rangle )$	Average burst size for X (Y)	$\langle {B}_{x}^{2}\rangle \,(\langle {B}_{y}^{2}\rangle )$	Second moment of burst size distribution for X (Y)

Based on the above discrete-state continuous-time Markov model, one can write a corresponding Chemical Master Equation (CME) that provides the time evolution of the joint probability density function *p*(*x*_*f*_, *x*_*b*_, *t*), for observing *x*_*f*_ free TF, and *x*_*b*_ bound TF molecules at time *t*^79,80^

2 $$\begin{array}{rcl}\frac{\partial p({x}_{f},{x}_{b},t)}{\partial t} & = & {k}_{x}\mathop{\sum }\limits_{i=0}^{{x}_{f}}{\alpha }_{x}(i)p({x}_{f}-i,{x}_{b},t)+{k}_{u}({x}_{b}+1)p({x}_{f}-1,{x}_{b}+1,t)\\  &  & +{k}_{b}({x}_{f}+1)(N-{x}_{b}+1)p({x}_{f}+1,{x}_{b}-1,t)\\  &  & +{\gamma }_{f}({x}_{f}+1)p({x}_{f}+1,{x}_{b},t)+{\gamma }_{b}({x}_{b}+1)p({x}_{f},{x}_{b}+1,t)\\  &  & -[{k}_{x}+{\gamma }_{f}{x}_{f}+{\gamma }_{b}{x}_{b}+{k}_{u}{x}_{b}+{k}_{b}{x}_{f}(N-{x}_{b})]p({x}_{f},{x}_{b},t).\end{array}$$

As the CME is analytically intractable, it is typically solved numerically through either the Finite State Projection algorithm^81–83^ or various Monte Carlo simulation techniques^84–88^. Taking an alternative approach, we focus on the statistical moments of the molecular counts and use the well-known Linear Noise Approximation^89–94^ to obtain closed-form formulas for the mean and noise levels.

In addition to the LNA, we assume that the binding/unbinding reactions are very fast compared to protein synthesis and degradations. This fast binding/unbinding limit, which is also known as quasi-static equilibrium or adiabatic limit, is common in the gene expression modelling^29,95,96^ and is supported by recent experiments^97^. This assumption simplifies our calculations to obtain the analytical expression for the Fano factors. As this limit implies negligible fluctuations due to binding kinetics, one may expect relaxing this limit can give rise to more variation in gene expression^95,97^. We have numerically verified that for our system, the qualitative behavior of results does not depend on this limit (see Fig. S1).

### Noise in free TF counts in the absence of decoy sites

When there are no decoys (*N* = 0), the moments of the free TF count can be solved exactly from the CME. In particular, the steady-state mean level $\overline{\langle {x}_{f,0}\rangle }$, and the Fano factor ${F}_{{x}_{f},0}$ (variance/mean) of *x*_*f*_(*t*) are given by^26,98^,

3 $$\overline{\langle {x}_{f,0}\rangle }=\frac{{k}_{x}\langle {B}_{x}\rangle }{{\gamma }_{f}},\,{\rm{and}}\,{F}_{{x}_{f},0}:=\frac{\overline{\langle {x}_{f,0}^{2}\rangle }-{\overline{\langle {x}_{f,0}\rangle }}^{2}}{\overline{\langle {x}_{f,0}\rangle }}=\frac{\langle {B}_{x}\rangle +\langle {B}_{x}^{2}\rangle }{2\,\langle {B}_{x}\rangle },$$

respectively, where 〈*B*_*x*_〉 and $\langle {B}_{x}^{2}\rangle$ are the first and second-order moments of the burst size *B*_*x*_. Throughout the paper we use angular brackets $\langle \,\rangle$ to denote the expected value operation, and $\overline{\langle \,\rangle }$ to represent the steady-state expected value. Note that the Fano factor is completely determined by the burst size distribution and is independent of the burst arrival rate and the protein decay rate. As expected, we recover the Poisson limit (${F}_{{x}_{f},0}=1$) for non-bursty production *B*_*x*_ = 1 with probability one, and noise is super-Poissonian (${F}_{{x}_{f},0} > 1$) for bursty production. In the special case where *B*_*x*_ follows a geometric distribution

4 $${\alpha }_{x}(i)={(1-1/\langle {B}_{x}\rangle )}^{i-1}/\langle {B}_{x}\rangle {\rm{for}}\,i\in \{1,2,3,\mathrm{..}.\},$$

the steady-state Fano factor ${F}_{{x}_{f},0}=\langle {B}_{x}\rangle$ is equal to the mean burst size.

### Bound TF’s degradation titrates the regulating activity of TF

In the presence of decoy (*N* > 0), exact solutions to the mean and noise levels are unavailable, and one has to resort to approximation techniques. Recall from (1), that the probability of the TF binding event is nonlinearly related to the molecular counts via the product term *x*_*f*_(*t*)*x*_*b*_(*t*). This nonlinearity results in unclosed moment dynamics – the time evolution of lower-order moments depends on higher-order moments^99,100^. For example, the dynamics of the means 〈*x*_*f*_〉, 〈*x*_*b*_〉 depends on the second order moment 〈*x*_*f*_*x*_*b*_〉 (see SI, section 1). Typically approximate closure schemes are employed to solve for moments in such cases^101–111^. Here, we use the Linear Noise Approximation (LNA) method, where assuming small fluctuations in *x*_*f*_(*t*) and *x*_*b*_(*t*) around their respective mean values 〈*x*_*f*_〉 and 〈*x*_*b*_〉, the nonlinear term is linearized as *k*_*b*_*x*_*f*_*x*_*b*_ ≈ *k*_*b*_(*x*_*f*_〈*x*_*b*_〉 + 〈*x*_*f*_〉*x*_*b*_ − 〈*x*_*b*_〉〈*x*_*f*_〉). Exploiting this linearization, the probability of all events in (1) become linear with respect to the molecular counts, resulting in closed moment dynamics (see SI, section 1). A direct result of using the LNA is that the time evolution of the means is identical to the deterministic chemical rate equations

5a $$\frac{d\langle {x}_{f}\rangle }{dt}={k}_{x}\langle {B}_{x}\rangle +{k}_{u}\langle {x}_{b}\rangle -{k}_{b}N\langle {x}_{f}\rangle +{k}_{b}\langle {x}_{b}\rangle \langle {x}_{f}\rangle -{\gamma }_{f}\langle {x}_{f}\rangle ,$$

5b $$\frac{d\langle {x}_{b}\rangle }{dt}=-{k}_{u}\langle {x}_{b}\rangle +{k}_{b}N\langle {x}_{f}\rangle -{k}_{b}\langle {x}_{b}\rangle \langle {x}_{f}\rangle -{\gamma }_{b}\langle {x}_{b}\rangle .$$

**Figure 2:**
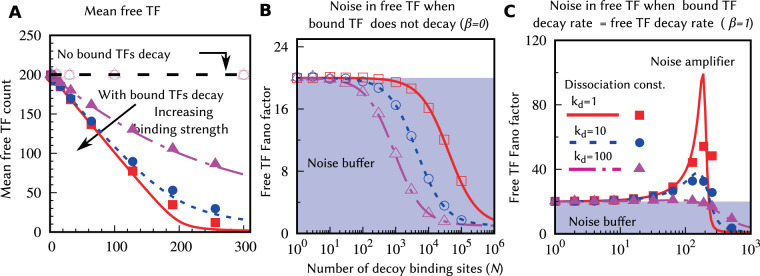
Degradation of bound TFs reduces the mean and drives non-monotonicity in the free TF noise level: The mean and the Fano factor for free TF counts are plotted against the total decoy binding sites *N* for different values of the dissociation constant (*k*_*d*_ = 1, *k*_*d*_ = 10 and *k*_*d*_ = 100). Lines are plotted using the analytical formulas (6) and (9), and the results with symbols are obtained from stochastic simulations using Gillespie algorithm [112]. (**A**) When bound TFs are protected from degradation, the mean becomes independent of *N* and *k*_*d*_. In the presence of bound TF degradation (results are shown for *β* = 1), the mean free TF count decreases with *N*. This decay becomes faster for larger binding strengths. (**B**) If bound TFs are protected from the degradation, the Fano factor decreases monotonically with *N*, suggesting decoys role as a noise buffer. (**C**) For intermediate values of decoy sites, a large noise enhancement can be seen in the presence of bound TF degradation. This noise amplification becomes larger for smaller values of *k*_*d*_. The Fano factors for both cases (**B**,**C**) approach to the Poissonian limit (Fano factor = 1) for large *N*. The parameters used for this figure: 〈*B*_*x*_〉 = 20, *k*_*x*_ = 10 *hr*^−1^, and *γ*_*f*_ = 1.0 *hr*^−1^ per protein molecule. For simulations, *k*_*b*_ = 50 *hr*^−1^ per pair of molecules.

Solving the above equations at steady state, and further considering fast binding/unbinding rates (*k*_*b*_ → ∞, *k*_*u*_ → ∞) for a given dissociation constant *k*_*d*_ = *k*_*u*_/*k*_*b*_, yields the following mean levels for the unbound and bound TF

6 $$\overline{\langle {x}_{f}\rangle }=\frac{\overline{\langle {x}_{f,0}\rangle }-N\beta -{k}_{d}+\sqrt{{(\overline{\langle {x}_{f,0}\rangle }-N\beta -{k}_{d})}^{2}+4{k}_{d}\overline{\langle {x}_{f,0}\rangle }}}{2},\,\overline{\langle {x}_{b}\rangle }=\frac{N\overline{\langle {x}_{f}\rangle }}{{k}_{d}+\overline{\langle {x}_{f}\rangle }},$$

respectively. Here, $\overline{\langle {x}_{f,0}\rangle }$ given by (3) is the mean TF count in the absence of decoy sites. When bound TFs are protected from degradation *β* = 0, the mean free TF count $\overline{\langle {x}_{f}\rangle }=\overline{\langle {x}_{f,0}\rangle }={k}_{x}\langle {B}_{x}\rangle /{\gamma }_{f}$ becomes independent of decoy numbers^42,52^. In contrast, with bound TF degradation *β* > 0, the mean free TF count monotonically decreases with increasing decoy numbers, with the decrease being faster for stronger binding affinity (or lower dissociation constant). This point is exemplified in Fig. 2(A) where we plot $\overline{\langle {x}_{f}\rangle }$ as a function of *N* for different dissociation constants. In the limit of a small number of decoys, (6) can be approximated as

7 $$\overline{\langle {x}_{f}\rangle }\approx \overline{\langle {x}_{f,0}\rangle }-\frac{\overline{\langle {x}_{f,0}\rangle }}{\overline{\langle {x}_{f,0}\rangle }+{k}_{d}}N\beta ,$$

and the rate of decrease of $\overline{\langle {x}_{f}\rangle }$ with increasing *N* is inversely proportional to *k*_*d*_. In the limit of large *N* and *β* > 0,

8 $$\overline{\langle {x}_{f}\rangle }\approx \frac{{k}_{d}\overline{\langle {x}_{f,0}\rangle }}{N\beta },$$

exhibiting a 1/*N* scaling of mean free TF levels with decoy abundance. Both these limits emphasize the point that when *β* > 0, increasing decoy numbers titrate away the TF, leading to reduced levels of the free TF. These results are consistent with experimental data in *Saccharomyces cerevisiae* showing reduced activity of the TF as a result of decoy binding^37^.

### Bound TF’s degradation enhances noise in the free TF count

Next, LNA is used to derive ${F}_{{x}_{f}}$, the steady-state Fano factor of the free TF level in the presence of decoys. Assuming fast binding and unbinding of TFs to decoy sites (*k*_*b*_ → ∞, *k*_*u*_ → ∞, fixed *k*_*d*_ = *k*_*u*_/*k*_*b*_) we obtain (see SI, section 1 for details)

9 $$\begin{array}{l}{F}_{{x}_{f}}=\frac{\overline{\langle {x}_{f}^{2}\rangle }-{\overline{\langle {x}_{f}\rangle }}^{2}}{\overline{\langle {x}_{f}\rangle }}=1+\frac{\overline{\langle {x}_{f}\rangle }[({F}_{{x}_{f},0}-1)\overline{\langle {x}_{f,0}\rangle }+\beta {f}^{2}N]}{(\overline{\langle {x}_{f,0}\rangle }-\beta {f}^{2}N)(Nf(1-f)+\overline{\langle {x}_{f}\rangle })},\,{\rm{with}}\,f=\frac{\overline{\langle {x}_{b}\rangle }}{N}=\frac{\overline{\langle {x}_{f}\rangle }}{{k}_{d}+\overline{\langle {x}_{f}\rangle }}.\end{array}$$

Here, ${F}_{{x}_{f},0}$ is the Fano factor in the absence of decoy binding sites (3), and *f* is the fraction of bound decoys. As expected, in the limit of no decoys (*N* → 0) or weakly binding decoys (*k*_*d*_ → ∞), ${F}_{{x}_{f}}\to {F}_{{x}_{f},0}$.

When bound TFs are protected from degradation (*β* = 0), then $\overline{\langle {x}_{f}\rangle }=\overline{\langle {x}_{f,0}\rangle }$ and the fraction of bound decoy *f* becomes independent of *N*. In this scenario, (9) simplifies to

10 $${F}_{{x}_{f}}=1+\frac{{F}_{{x}_{f},0}-1}{\frac{Nf(1-f)}{\overline{\langle {x}_{f,0}\rangle }}+1},\,{\rm{w}}{\rm{i}}{\rm{t}}{\rm{h}}\,f=\frac{\overline{\langle {x}_{f,0}\rangle }}{{k}_{d}+\overline{\langle {x}_{f,0}\rangle }}.$$

As *f* is independent of *N*, it is clear from (10) that ${F}_{{x}_{f}}$ monotonically decreases from ${F}_{{x}_{f},0}$ to 1 with increasing *N* (Fig. 2(B)). Thus, when the bound TFs are protected from degradation, the decoy sites function as a *noise buffer* with ${F}_{{x}_{f}} < {F}_{{x}_{f},0}$^52^.

An interesting finding that emerges form (9) is that when *β* > 0, ${F}_{{x}_{f}}$ can vary non-monotonically with *N*. This point is illustrated in Fig. 2(C), where we plot ${F}_{{x}_{f}}$ as a function of decoy abundance for *β* = 1. While ${F}_{{x}_{f}}$ monotonically decreases to 1 for weak binding affinities (similar to the case of *β* = 0), for strong binding affinities ${F}_{{x}_{f}}$ first increases with increasing *N* to reach a maxima, before decreasing back to the Poisson limit for large *N*. In essence, with degradation of bound TFs, decoys can function as a *noise amplifier* (${F}_{{x}_{f}} > {F}_{{x}_{f},0}$). Checking the sign of the derivative $d{F}_{{x}_{f}}/dN > 0$ in the limit *N* → 0 leads to an analytical condition for noise enhancement – if the dissociation constant is below a critical threshold

11 $${k}_{d} < {k}_{d}^{th}=\frac{{F}_{{x}_{f},0}\beta \overline{\langle {x}_{f,0}\rangle }}{{F}_{{x}_{f},0}-1},$$

then the Fano factor will increase starting from ${F}_{{x}_{f},0}$ as decoy sites are introduced. For ${F}_{{x}_{f},0}\gg 1$, the threshold value simplifies to ${k}_{d}^{th}=\beta \overline{\langle {x}_{f,0}\rangle }$, independent of ${F}_{{x}_{f},0}$. It turns out that this condition for noise enhancement implies that the fraction of bound decoys $f\mathrm{ > 1/(1}+\beta )$ when a small number of decoys are present. In spite of the noise amplification, it is important to point out that ${F}_{{x}_{f}}\to 1$ as *N* → ∞ irrespective of the value of *β*. Thus, strongly-binding decoys that function as a noise amplifier for small *N*, become a noise buffer for large *N*.

Overall these results suggest that decoys buffer noise in a variety of scenarios: *β* = 0 (irrespective of *N* and *k*_*d*_), or for a large number of decoys (irrespective of *β* and *k*_*d*_) or if decoys have sufficiently weak binding affinities (irrespective of *β* and *N*). In contrast, decoys function as a noise amplifier when *β* > 0 provided two other conditions hold – decoys have sufficiently strong binding affinities as per (11) *and* are not present in large numbers.

To check the validity of the linearization and fast binding/unbinding approximations, we perform kinetic Monte Carlo simulations using the Gillespie algorithm^112^ to obtain numerically exact results. In Fig. 2, along with the analytical results (lines) we also plot the simulation results (symbols). The match between analytical and simulations results are quite well, especially for large and intermediate *k*_*d*_ values. For small *k*_*d*_ values with *β* ≠ 0, there is a clear deviation from the analytical results. In this regime, being fluctuations very large, the linearization approximation based on absence of large fluctuations may not be justified. However, as can be seen, the qualitative features do not depend on these approximations.

**Figure 3:**
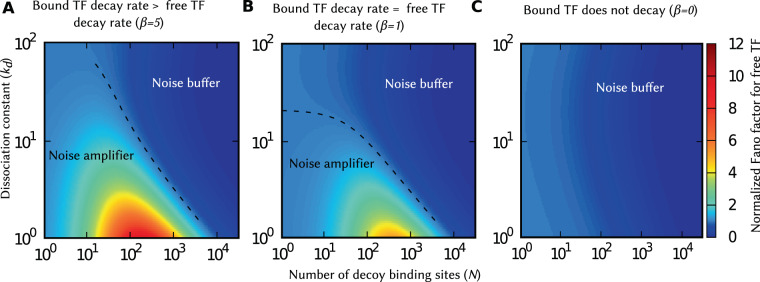
Decreasing stability of bound TFs expands the parameter space for decoy-mediated noise enhancement. The normalized Fano factors (${F}_{{x}_{f}}/{F}_{{x}_{f},0}$) for different degradation rates of bound TFs are plotted as a function of decoy numbers (*N*) and the dissociation constant (*k*_*d*_), for a constant mean free TF count. The color box represents the scale for the normalized Fano factor, and its value larger than one implies noise enhancement. For smaller values of *k*_*d*_ and *N*, the decoy acts as a noise amplifier when bound TFs are unstable. The region of noise enhancement (demarcated by a dashed line representing ${F}_{{x}_{f}}={F}_{{x}_{f},0}$) increases with increasing degradation rate of bound TFs. Parameters used: $\overline{\langle {x}_{f}\rangle }=20$ molecules, 〈*B*_*x*_〉 = 20, and *γ*_*f*_ = 1 *hr*^−1^ per protein molecule. These plots are generated by using (9). While to keep $\overline{\langle {x}_{f}\rangle }$ constant, we change $\overline{\langle {x}_{f,0}\rangle }$ accordingly by varying *k*_*x*_ and obeying (6).

As illustrated in Fig. 2(A), bound TFs degradation reduces the number of available TFs with increasing decoy abundance. This naturally leads to the question: is the noise behavior reported in Fig. 2(C) also seen when the mean free TF count is held constant at given desired level? In Fig. 3, we plot the noise as a function *N* and *k*_*d*_ for a given mean free TF count $\overline{\langle {x}_{f}\rangle }$ by simultaneously enhancing the production rate *k*_*x*_ as per (6). Our results show similar qualitative behaviors with decoys functioning as a noise buffer for *β* = 0 (Fig. 3(C)), becoming a noise amplifier when *β* = 1 for sufficiently small *N* and *k*_*d*_ (Fig. 3(B)). Interestingly, the region of noise amplification is greatly enhanced when bound TFs become more unstable compared to their free counterparts (Fig. 3(A)).

### Noise in free TF counts in a mixture of strong and weak decoy binding sites

Inside cells, TFs bind to various decoy sites with different affinities^33^. How do fluctuations in the TF count gets affected by a mixture of nonidentical decoys? To address this question, we study a system two decoy types D1 and D2 that are present in numbers *N*_1_ and *N*_2_, respectively. We assume that each free TF molecule can bind to both decoy types with the same (diffusion-limited) rate *k*_*b*_, but unbinds with different rates *k*_*u*1_ and *k*_*u*2_, respectively, leading to two different dissociation constants *k*_*d*1_ = *k*_*u*1_/*k*_*b*_ and *k*_*d*2_ = *k*_*u*2_/*k*_*b*_. As before, TFs are synthesized in random bursts, the free and bound TFs decay with rates *γ*_*f*_ and *γ*_*b*_ (the decay rates of TFs bound to D1 and D2 are assumed to be equal). Together with (1a) and (1d), the stochastic model is now governed by the following probabilities representing jumps in the population counts in the infinitesimal time interval (*t*, *t* + *dt*]

12a $$\begin{array}{c}{\rm{TF}}\,{\rm{binding}}\,{\rm{decoy}}\,{\rm{D1}}:{\rm{Probability}}\{{x}_{f}(t+dt)={x}_{f}(t)-1,{x}_{b1}(t+dt)={x}_{b1}(t)+1\}={k}_{b}{x}_{f}(t)({N}_{1}-{x}_{b1}(t))dt,\end{array}$$

12b $$\begin{array}{c}{\rm{T}}{\rm{F}}\,{\rm{b}}{\rm{i}}{\rm{n}}{\rm{d}}{\rm{i}}{\rm{n}}{\rm{g}}\,{\rm{d}}{\rm{e}}{\rm{c}}{\rm{o}}{\rm{y}}\,{\rm{D}}2:{\rm{P}}{\rm{r}}{\rm{o}}{\rm{b}}{\rm{a}}{\rm{b}}{\rm{i}}{\rm{l}}{\rm{i}}{\rm{t}}{\rm{y}}\{{x}_{f}(t+dt)={x}_{f}(t)-1,{x}_{b2}(t+dt)={x}_{b2}(t)+1\}={k}_{b}{x}_{f}(t)({N}_{2}-{x}_{b2}(t))dt,\end{array}$$

12c $$\begin{array}{c}{\rm{T}}{\rm{F}}\,{\rm{u}}{\rm{n}}{\rm{b}}{\rm{i}}{\rm{n}}{\rm{d}}{\rm{i}}{\rm{n}}{\rm{g}}\,{\rm{f}}{\rm{r}}{\rm{o}}{\rm{m}}\,{\rm{D}}1:{\rm{P}}{\rm{r}}{\rm{o}}{\rm{b}}{\rm{a}}{\rm{b}}{\rm{i}}{\rm{l}}{\rm{i}}{\rm{t}}{\rm{y}}\{{x}_{f}(t+dt)={x}_{f}(t)+1,{x}_{b1}(t+dt)={x}_{b1}(t)-1\}={k}_{u1}{x}_{b1}(t)dt,\end{array}$$

12d $$\begin{array}{c}{\rm{T}}{\rm{F}}\,{\rm{u}}{\rm{n}}{\rm{b}}{\rm{i}}{\rm{n}}{\rm{d}}{\rm{i}}{\rm{n}}{\rm{g}}\,{\rm{f}}{\rm{r}}{\rm{o}}{\rm{m}}\,{\rm{D}}2:{\rm{P}}{\rm{r}}{\rm{o}}{\rm{b}}{\rm{a}}{\rm{b}}{\rm{i}}{\rm{l}}{\rm{i}}{\rm{t}}{\rm{y}}\{{x}_{f}(t+dt)={x}_{f}(t)+1,{x}_{b2}(t+dt)={x}_{b2}(t)-1\}={k}_{u2}{x}_{b2}(t)dt,\end{array}$$

12e $${\rm{D1}}-{\rm{bound}}\,{\rm{TF}}\,{\rm{degradation}}:{\rm{Probability}}\{{x}_{b1}(t+dt)={x}_{b1}(t)-1\}={\gamma }_{b}{x}_{b1}(t)dt,$$

12f $${\rm{D2}}-{\rm{bound}}\,{\rm{TF}}\,{\rm{degradation}}:{\rm{Probability}}\{{x}_{b2}(t+dt)={x}_{b2}(t)-1\}={\gamma }_{b}{x}_{b2}(t)dt.$$

Here *x*_*b*1_ and *x*_*b*2_ denote the number TFs bound to decoys D1 and D2, respectively. We apply the linear noise approximation to obtain time evolution of statistical moments (presented in the SI, section 2), and solve the resulting moment equations at the steady to compute the Fano factor numerically as the analytical formula for the noise level becomes quite involved.

**Figure 4:**
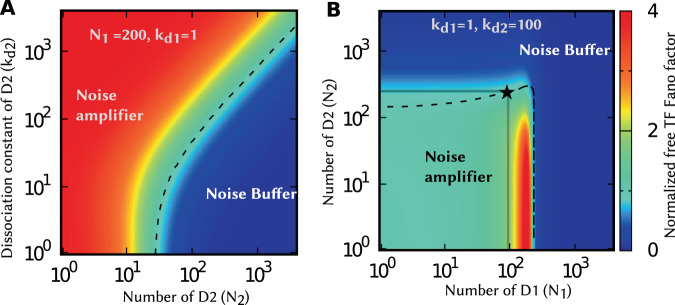
Noise-buffering decoys can mitigate the effects of noise-amplifying decoys. (**A**) The density plots of normalized Fano factor against *N*_2_ and *k*_*d*2_ for *N*_1_ = 200 and *k*_*d*1_ = 1. (**B**) The normalized Fano factor against *N*_1_ and *N*_2_ for *k*_*d*1_ = 1 and *k*_*d*2_ = 100. The noise enhancement region is separated from noise buffer region with dashed lines (correspond to ${F}_{{x}_{f}}/{F}_{{x}_{f},0}=1$). While individual decoy types act oppositely on free TF noise, presence of both can cancel this effect and maintain the same noise level as in the absence of any decoys. The point marked by the star is such a representative point. Parameter used: 〈*B*_*x*_〉 = 20, *γ*_*f*_ = *γ*_*b*_ = 1 *hr*^−1^ per molecule, *k*_*x*_ = 10 *hr*^−1^, and *k*_*b*_ = 1000 *hr*^−1^.

Noise in the free TF counts is investigated in two complementary ways. In Fig. 4A, we fix the number of decoys D1 and their binding affinity such that D1 itself functions as a noise amplifier. The noise in the free TF counts is then plotted as a function of the number of decoys D2 and its binding affinity. Recall that the dashed line represents ${F}_{{x}_{f}}={F}_{{x}_{f},0}$, i.e., the noise with decoys is the same as the noise in the absence of decoys. Having a large number of decoys D2 makes the overall decoy mixture a noise buffer, with the dashed line showing how large of a pool *N*_2_ is needed as a function of *k*_*d*2_. In Fig. 4B we fix the binding affinity of both decoys and plot the noise level as a function of decoys abundances. Here the noise enhancement is only observed when both decoys are present at small numbers, and the decoy mixture is a noise buffer even if one of the decoys is present in sufficiently large numbers. It is interesting to note that in Fig. 4B, D1 by itself is a noise amplifier (gray line intersection with the x-axis), D2 by itself is a noise buffer (gray line intersection with the y-axis), but their combined presence (star on the dashed line) mitigates each other’s effect, and the noise level is similar to when there were no decoys.

### Quantifying noise propagation from TF to downstream target proteins

Having quantified the magnitude of fluctuations in the free TFs counts, we next focus our attention on the time-scale of fluctuations. Given that the available TF pool activates downstream target proteins, the time scale at which fluctuations relax back to mean levels is key in understanding downstream noise propagation^113,114^. For example, for a given noise level, more prolonged fluctuations in the free TF counts will lead to higher noise in the expression of the target protein.

**Figure 5:**
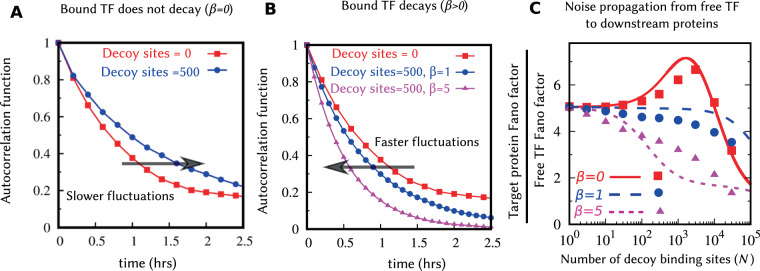
Bound TFs degradation leads to faster fluctuations in the free TF counts reducing noise propagation to downstream proteins. The simulation results of autocorrelation function *R*(*τ*) of the free TF count given by (13) for (**A**) Bound TFs are protected from degradation, and (**B**) Bound TFs are subject to degradation. The addition of decoy binding sites makes the autocorrelation decay slower in (**A**) but faster in (**B**). (**C**) Noise propagation from the free TF to the downstream target protein is measured by the ratio of the Fano factors ${F}_{y}/{F}_{{x}_{f}}$. Lines are the plotted using the analytical formula of *F*_*y*_ and ${F}_{{x}_{f}}$, given by (16) and (9) respectively. Symbols represent corresponding results with simulations. Noise propagation is enhanced when *β* = 0 consistent with the shift in the autocorrelation function to the right in Fig. 5A. In contrast, noise propagation is reduced when *β* > 0. Parameters used: $\overline{\langle {x}_{f}\rangle }=20$ molecules, 〈*B*_*x*_〉 = 20, *k*_*d*_ = 1, 〈*B*_*y*_〉 = 1, *k*_*y*_ = 10 *hr*^−1^, and *γ*_*f*_ = *γ*_*y*_ = 1 *hr*^−1^ per protein molecule. For simulations, *k*_*b*_ = 50 per pair of molecules. To keep $\overline{\langle {x}_{f}\rangle }$ constant, we change $\overline{\langle {x}_{f,0}\rangle }$ accordingly by varying *k*_*x*_ and obeying (6).

The time-scale of fluctuations is characterized using the autocorrelation function defined as,

13 $$R(\tau )=\frac{\langle {x}_{f}(\tau +t){x}_{f}(t)\rangle -{\overline{\langle {x}_{f}\rangle }}^{2}}{\overline{\langle {x}_{f}^{2}\rangle }-{\overline{\langle {x}_{f}\rangle }}^{2}},$$

where time *t* is sufficiently large for the system to reach the steady-state. In the absence of any decoy binding sites, the decay in the autocorrelation function is given by exp(−*γ*_*f*_*τ*)^52^. Note that this function only depends on the decay rate and independent of bursting parameters. Thus, the magnitude and time-scale of fluctuations can be independently tuned using (3) and (13) via the burst size and the decay rate. In the presence of decoy binding sites, we compute *R*(*τ*) numerically from stochastic realizations of *x*_*f*_(*t*) obtained via kinetic Monte Carlo simulations^112^. Figure 5 plots the decay of *R*(*τ*) as a function of lag-time *τ*, in the absence and presence of bound TF degradation. When the bound TFs are protected from degradations, the autocorrelation function shifts to the right leading to slower and more prolonged fluctuations in the free TF count. In contrast, when bound TFs are unstable, *R*(*τ*) shifts to the left, resulting in faster fluctuations, and the shift is more pronounced for larger values of *β*.

To understand how fluctuations in the free TF propagate downstream, we consider a case where the stochastic synthesis of target protein *Y* is activated by available TFs (see Fig. 1). Instead of incorporating the direct binding of TFs to the promoter of the target gene, we model the activation via a linear dose-response by the TF — the synthesis rate of *Y* is proportional to the number of available TF. We note that this linearity assumption is more appropriate when the binding affinity of TFs to the target gene is relatively small. This limit provides a theoretical understanding of noise propagation in the target protein as the mathematical formula for the noise can be derived.

The target protein is assumed to be produced in bursts *B*_*y*_ with an arbitrary burst size distribution

14 $${\rm{Probability}}\{{B}_{y}=i\}={\alpha }_{y}(i),$$

and burst frequency *x*_*f*_*k*_*y*_ that increases linearly with the free TF count. The probabilistic events governing the production and decay of target proteins are given by

15a $${\rm{Probability}}\{y(t+dt)=y(t)+i\}={k}_{y}{x}_{f}{\alpha }_{y}(i)dt,$$

15b $${\rm{Probability}}\{y(t+dt)=y(t)-1\}={\gamma }_{y}y(t)dt,$$

where *y*(*t*) is the population count of protein *Y* at time *t*. Applying LNA to the stochastic model (1) and (15) yields the following expression for the steady-state Fano factor of *y*(*t*) in the limit of fast binding/unbinding (*k*_*b*_ → ∞, *k*_*u*_ → ∞ with *k*_*d*_ = *k*_*u*_/*k*_*b*_ being finite),

16 $$\begin{array}{c}{F}_{y}=\frac{\overline{\langle {y}^{2}\rangle }-{\overline{\langle y\rangle }}^{2}}{\overline{\langle y\rangle }}\,=\frac{\langle {B}_{y}^{2}\rangle +\langle {B}_{y}\rangle }{2\langle {B}_{y}\rangle }+\frac{{F}_{{x}_{f},0}\overline{\langle y\rangle }\,\overline{\langle {x}_{f,0}\rangle }{\gamma }_{y}}{(\overline{\langle {x}_{f,0}\rangle }-\beta {f}^{2}N)[(Nf(1-f)+\overline{\langle {x}_{f}\rangle }){\gamma }_{y}+(\overline{\langle {x}_{f,0}\rangle }-\beta {f}^{2}N){\gamma }_{f}]},\end{array}$$

where $\overline{\langle y\rangle }=\overline{\langle {x}_{f}\rangle }{k}_{y}\langle {B}_{y}\rangle /{\gamma }_{y}$ is the steady-state mean level of the target protein, 〈*B*_*y*_〉 is the average burst size, and $\langle {B}_{y}^{2}\rangle$ is the second-order moment of the burst size. Note that this noise level is made up of two components – the first component on the right-hand-side of (16) represents the noise from bursting of the target protein, and the second component is the noise in *Y* due to upstream fluctuations in the free TF count. In the absence of any decoy, (16) reduces to

17 $${F}_{y,0}=\frac{\langle {B}_{y}^{2}\rangle +\langle {B}_{y}\rangle }{2\langle {B}_{y}\rangle }+\frac{{F}_{{x}_{f},0}\langle {B}_{y}\rangle {k}_{y}}{{\gamma }_{y}+{\gamma }_{f}}.$$

Our analysis shows that when bound TFs are stable (*β* → 0), the second noise component in *F*_*y*_ monotonically decreases to zero with increasing decoy numbers. For *β* > 0, similar to the free TF count, noise enhancement in the target protein occurs for a small number of high-affinity decoys (see Fig. S2). However, both the magnitude (*F*_*y*_/*F*_*y*,0_)and the region of the noise enhancement are smaller compared to that of the free TF count (see Fig. S2 and compare with Fig. 3).

Noise propagation can be measured by the ratio of the target protein noise to the free TF noise, ${F}_{y}/{F}_{{x}_{f}}$. Figure 5C shows the noise propagation as a function of decoy numbers for different decay rates of bound TF. Here the y-axis intercept quantifies noise propagation in the absence of decoys. When the bound TFs are protected from degradation, noise propagation is first enhanced as expected from the right-shift of the autocorrelation function (Fig. 5A), but them sharply decreases at higher decoy abundances. Note that the increase in noise propagation seen at intermediate decoy numbers does not imply an increase in the target protein noise level, as at the same time the free TF noise level decreases with increasing *N* for *β* = 0 (Fig. 3). When bound TFs are unstable, noise propagation is reduced (Fig. 5C) due to faster time-scale of fluctuations of the free TF count (Fig. 5B). This implies that the noise increase seen in the free TF level when *β* > 0 (Fig. 3) is buffered by a decreased noise propagation, and hence, the region of noise enhancement for the target protein is reduced compared to that of the free TF count. The qualitative feature of noise propagation agrees with the stochastic simulations results. However, the quantitative match between stochastic simulations and the LNA results is poor for *β* > 0 (Fig. 5C).

## Discussion

The sequestration of transcription factors by genomic decoy sites can either protect it from degradation^43,44^ or make it more facile for degradation^45^. Here, we have systematically investigated how the magnitude and time-scale of TF copy-number fluctuations are impacted by the stability of the bound TF, the number and affinity of decoy sites. While this contribution focuses on TFs binding to genomic decoy sites, these results are applicable to other classes of proteins, for example, RNA-binding proteins binding to sites on RNA^115–118^.

**Figure 6:**
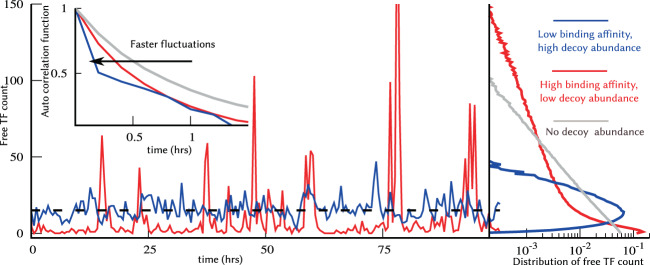
High-affinity low-abundance decoys, and low-affinity high-abundance decoys result in the same mean free TF counts with contrasting fluctuation dynamics. Stochastic realizations of the number of free TFs for high-affinity low-abundance decoys (red), and low-affinity high-abundance decoys (blue) when *β* = 1, along with their corresponding probability distributions on the right. Both scenarios yield the same average number of free TF molecules, but high-affinity decoys drive significantly enhanced noise levels. The instability of the bound TF leads to faster time-scale of fluctuations as illustrated by the left-shift of the autocorrelation function (inset). For comparison, the free TF count distribution and the autocorrelation function for the no-decoy case are also shown with grey lines. The parameters used are as follows: 〈*B*_*x*_〉 = 20, *k*_*x*_ = 10 *hr*^−1^, *γ*_*f*_ = *γ*_*b*_ = 1.0 *hr*^−1^ per protein molecule and *k*_*b*_ = 50 *hr*^−1^ per pair of molecules. For high affinity decoy *k*_*d*_ = 1 and *N* = 245, and low affinity decoy *k*_*d*_ = 100 and *N* = 1400. This choice produces the same mean TF count for both the cases, $\overline{\langle {x}_{f}\rangle }\simeq 15$. For the no-decoy case: *k*_*x*_ = 0.75 *hr*^−1^ to keep $\overline{\langle {x}_{f}\rangle }\simeq 15$.

Our results show that the degradation of decoy-bound TFs critically impacts both the mean and noise levels of the free TF pool. More specifically, while the average number of free (available) TFs monotonically decreases with increasing decoy abundance, the noise levels can sharply increase at low/intermediate decoy numbers before attenuating to Poisson levels as *N* → ∞ (Fig. 2). This behavior can be contrasted to when bound TFs are protected from degradation, in which case the mean free TF count becomes invariant of *N*, and decoys always buffer noise (Figs. 2 and 3). When *β* > 0, high-affinity decoys can transition from being noise amplifiers to noise buffers as their numbers are increased (Fig. 2). This point is exemplified in Fig. 6 where high-affinity low-abundance decoys and low-affinity high-abundance decoys result in the same average number of freely available TFs, but with much higher noise in the former case. Moreover, a mixture of both decoy types can mitigate each other’s effect leading to no noise enhancement or buffer (Fig. 4).

Closed-form formulas for the noise levels derived using the Linear Noise Approximation led to precise conditions for noise enhancement – the number of decoys is not large, and their binding affinity is strong enough such that the fraction of bound decoys is higher than 1/(*β* +1 ). For example, *β* = 1 for a stable TF whose turnover is primarily governed by dilution from cell growth, and this condition implies that more than 50% decoy sites must be occupied. The decoy-mediated noise enhancement is higher for large values of *β* and for more “sticky” or higher affinity decoys (Fig. 3). It is interesting to point out that the peak noise enhancement generally occurs when the number of decoy sites is comparable to the TF counts when both bound and free TFs decay with the same rate. For example, in Fig. 2C there is an average of 200 TF molecules in the absence of decoys and the noise peak is also seen around *N* ≈ 200. Note that our results are restricted to TF production in stochastic bursts, and it remains to be seen if these results generalize to other noise mechanisms in gene expression, such as, extrinsic noise that arises from intercellular differences in cell size and the abundance of expression machinery^98,119–122^.

The speed of fluctuation in free TF counts also shows distinct features in the presence and absence of bound TF degradation. While the fluctuations become faster in the presence of bound TF degradation, as indicated by the left-shift of the autocorrelation function (Fig. 5), we see an opposite result when bound TFs do not degrade. These results have important implications on how noise propagates from the TF to downstream target proteins. For example, when *β* > 0 decoys can amplify noise in the free TF levels, while at the same time make fluctuations in TF counts relax back faster attenuating downstream noise transmission. As a result of these two opposing forces, the noise in the target protein may not increase even though the noise in the free TF level has increased. This can be seen for *β* = 5 in Fig. 3 where for *N* = 100 and *k*_*d*_ = 10 the decoy is a noise amplifier, but as seen in Fig. S2, for the exact same parameter values the noise in the target protein has reduced compared to the no-decoy case.

The analytical formulas for the noise give theoretical insights into the role decoys on the noise enhancement. These are derived by linearizing the propensity for the binding event assuming small copy number fluctuations around the statistical mean and then taking fast bind/binding limit. Using numerical exact stochastic simulations, we have shown that the main results are good agreement with the theory. The quantitative match between theory and simulations can be poor where the fluctuations are large. This happens for higher decoy binding affinities and degradation rates for bound TFs. We numerically have found that results are not much sensitive to fast binding/unbinding limit (Fig. S1). The objective of obtaining more accurate theoretical results is a matter of future work.

Our novel finding of the dual role of decoys as noise amplifiers/buffers encourages more investigation into the regulatory function of decoys in complex gene networks. In our study, we have considered target gene dose-response is linear. In future, we want to investigate the role of decoy sites in gene networks that contain nonlinearity. For example, in genetic/signalling circuits with oscillatory dynamics decoys can tune the oscillation frequency^48^, enhance coherence^47^, and it will be interesting to see if decoys can make biological clocks more robust to molecular noise. An exciting avenue of experimental research is to use decoys as manipulations of phenotypic heterogeneity. For example, aberrant expression of resistance markers in individual melanoma cells have been shown to be a driver of cancer drug resistance^5^, and decoy-mediated noise buffering can play a therapeutic role in reducing such outlier cells. In the context of the Human Immunodeficiency Virus (HIV), noisy expression of a key viral protein, Tat, controls the cell-fate outcome between active viral replication and latency^16,123–126^. Latency is a dormant state of the virus and considered the biggest challenge in eradicating the virus from a patient since latent cells are long-lived and resistant to drug therapy^127^. Recently, small-molecule compounds have been identified that enhance noise in Tat expression for efficient reactivation of latent cells^128^, and here the role of decoys as noise amplifiers may allow for a Tat-specific compound-free approach for latency reversal.

## Supplementary information

Supplementary Information.
