# Supplementary material for: Enhancement of gene expression noise from transcription factor binding to genomic decoy sites
Source: Sci Rep. 2020 Jun 4;10:9126. doi: 10.1038/s41598-020-65750-2 (PMC7272470; doi:10.1038/s41598-020-65750-2)
Supplement: Supplementary file 1 — Supplementary Information. [file 41598_2020_65750_MOESM1_ESM.pdf]

# Supplementary Information for
# "Enhancement of gene expression noise from transcription binding to genomic decoy sites"

Supravat Dey[1,*], Mohammad Soltani[1], and Abhyudai Singh[1,2,3,4,*]

[1]Department of Electrical and Computer Engineering, University of Delaware, Newark, DE 19716, USA
[2]Department of Biomedical Engineering, University of Delaware, Newark, DE 19716, USA
[3]Department of Mathematical Sciences, University of Delaware, Newark, DE 19716, USA
[4]Center for Bioinformatics and Computational Biology, University of Delaware, Newark, DE 19716, USA
[*]supravat.dey@gmail.com and absingh@udel.edu

## 1. Dynamical equations for moments for free and bound TFs and target proteins

Let $\phi(x_f, x_b)$ be an arbitrary differentiable function of free TF count $x_f$, bound TF count $x_b$. The time evolution of $\langle \phi(x_f, x_b)\rangle$ obeying the chemical master equation Eq. (2) in the main text is given by,

$$\begin{aligned}\frac{d\langle \phi(x_f,x_b)\rangle}{dt} = \; & \langle k_x \textstyle\sum_{i=0}^{\infty} \alpha_x(i)\,[\phi(x_f+i,x_b)-\phi(x_f,x_b)]\rangle + \langle \gamma_f x_f\,[\phi(x_f-1,x_b)-\phi(x_f,x_b)]\rangle \\ & + \langle \gamma_b x_b\,[\phi(x_f,x_b-1)-\phi(x_f,x_b)]\rangle + \langle k_u x_b\,[\phi(x_f+1,x_b-1)-\phi(x_f,x_b)]\rangle \\ & + \langle k_b x_f (N-x_b)\,[\phi(x_f-1,x_b+1)-\phi(x_f,x_b)]\rangle. \end{aligned} \tag{S1}$$

The lower and upper limit of the summation is the minimum and the maximum copy number of the TF released during burst. Note that the last term (due to binding event) is nonlinear. This makes the moment dynamics unclosed in the sense that the dynamical equation of a given moment depends on the higher order moments. For example, the time evolution of $\langle x_f\rangle$, which can be obtained by substituting $\phi(x_f, x_b)$ with $x_f$ in (S1), is given by,

$$\frac{d\langle x_f\rangle}{dt} = k_x\langle B_x\rangle + k_u\langle x_b\rangle - \gamma_f\langle x_f\rangle - k_b N\langle x_f\rangle + k_b\langle x_b x_f\rangle. \tag{S2}$$

This dynamical equation of first order moment depends higher order moment $\langle x_b x_f\rangle$. Similarly, the dynamical equation of $\langle x_b x_f\rangle$ depends on the higher order moments such as $\langle x_b^2 x_f\rangle$ and $\langle x_b x_f^2\rangle$. To get closed form moment equations, we use the Linear Noise Approximation (LNA). Under this approximation, we linearise the nonlinear part of the binding term $k_b x_b x_f$ around the mean levels $\langle x_f\rangle$ and $\langle x_b\rangle$: $k_b x_b x_f = k_b(\langle x_b\rangle x_f + x_b\langle x_f\rangle - \langle x_b\rangle\langle x_f\rangle)$, assuming small fluctuations in copy numbers. Under the LNA, the

first order moment dynamics of $x_f$ and $x_b$ are given by,

$$\frac{d\langle x_f\rangle}{dt} = k_x\langle B_x\rangle + k_u\langle x_b\rangle - \gamma_f\langle x_f\rangle - k_b(N-\langle x_b\rangle)\langle x_f\rangle, \tag{S3a}$$

$$\frac{d\langle x_b\rangle}{dt} = -k_u\langle x_b\rangle - \gamma_b\langle x_b\rangle + k_b(N-\langle x_b\rangle)\langle x_f\rangle, \tag{S3b}$$

The steady value of the mean free TF and bound TF counts are obtained by solving the (S3) by setting time derivatives to zero:

$$\overline{\langle x_b\rangle} = \frac{N\overline{\langle x_f\rangle}}{k_d + \overline{\langle x_f\rangle} + \gamma_b/k_b}, \tag{S4a}$$

$$\overline{\langle x_f\rangle} = \frac{1}{2}\left[\overline{\langle x_{f,0}\rangle} - k_d - N\beta - \frac{\gamma_b}{k_b} + \sqrt{\left(\overline{\langle x_{f,0}\rangle} - k_d - N\beta - \frac{\gamma_b}{k_b}\right)^2 + 4\left(k_d + \frac{\gamma_b}{k_b}\right)\overline{\langle x_{f,0}\rangle}}\right], \tag{S4b}$$

where $\overline{\langle x_{f,0}\rangle} = k_x\langle B_x\rangle/\gamma_f$, $k_d = k_u/k_b$ and $\beta = \gamma_b/\gamma_f$. In the limit of large binding/unbinding rates ($k_b \to \infty$, $k_u \to \infty$ with $k_u/k_b = k_d$), the above expression of $\overline{\langle x_b\rangle}$ and $\overline{\langle x_f\rangle}$ reduce to,

$$\overline{\langle x_b\rangle} = \frac{N\overline{\langle x_f\rangle}}{k_d + \overline{\langle x_f\rangle}}, \tag{S5a}$$

$$\overline{\langle x_f\rangle} = \frac{1}{2}\left[\overline{\langle x_{f,0}\rangle} - k_d - N\beta + \sqrt{\left(\overline{\langle x_{f,0}\rangle} - k_d - N\beta\right)^2 + 4k_d\overline{\langle x_{f,0}\rangle}}\right], \tag{S5b}$$

as presented in the main text.

To obtain the unclosed dynamics for second moments, we use the same linearization scheme as describe above. Linearizing the binding term around their mean values we get,

$$\begin{aligned}\frac{d\langle x_f^2\rangle}{dt} =& k_x\langle B_x^2\rangle + \langle x_f\rangle\left[2k_x\langle B_x\rangle + k_b(N-\langle x_b\rangle - 2\langle x_b\rangle\langle x_f\rangle) + \gamma_f\right] + k_u\langle x_b\rangle + 2\langle x_b x_f\rangle(k_u + k_b\langle x_f\rangle) \\ & - 2\langle x_f\rangle^2\left[k_b(N-\langle x_b\rangle) + \gamma_f\right],\end{aligned} \tag{S6a}$$

$$\frac{d\langle x_b^2\rangle}{dt} = \langle x_b\rangle\left[k_u + 2k_b\langle x_b\rangle\langle x_f\rangle + \gamma_b\right] + \langle x_f\rangle k_b(N-\langle x_b\rangle) + 2k_b\langle x_b x_f\rangle(N-\langle x_b\rangle) - 2\langle x_b^2\rangle(k_u + k_b\langle x_f\rangle + \gamma_b), \tag{S6b}$$

$$\begin{aligned}\frac{d\langle x_b x_f\rangle}{dt} =& \langle x_b\rangle\left[-k_u + k_x\langle B_x\rangle\right] - k_b\langle x_f\rangle\left[(N-\langle x_b\rangle) + \langle x_b\rangle^2 - \langle x_b\rangle\langle x_f\rangle\right] \\ & - \langle x_b x_f\rangle\left[k_u + k_b(N-\langle x_b\rangle + \langle x_f\rangle) + \gamma_b + \gamma_f\right] + k_b\langle x_f^2\rangle(N-\langle x_b\rangle) + \langle x_b^2\rangle(k_b\langle x_f\rangle + k_u).\end{aligned} \tag{S6c}$$

In the case of downstream protein ($Y$), the additional first and second order moment dynamics are given by,

$$\frac{d\langle y\rangle}{dt} = k_y\langle B_y\rangle\langle x_f\rangle - \gamma_y\langle y\rangle, \tag{S7a}$$

$$\frac{d\langle y^2\rangle}{dt} = k_y\langle B_y^2\rangle\langle x_f\rangle + \gamma_y\langle y\rangle + 2k_y\langle B_y\rangle\langle x_f y\rangle - 2\gamma_y\langle y^2\rangle, \tag{S7b}$$

$$\frac{d\langle x_f y\rangle}{dt} = \langle y\rangle(k_x\langle B_x\rangle - k_b\langle x_b\rangle\langle x_f\rangle) + k_y\langle B_y\rangle\langle x_f^2\rangle + \langle x_b y\rangle(k_u + k_b\langle x_f\rangle) - \langle x_f y\rangle\left[k_b(N-\langle x_b\rangle) + \gamma_f + \gamma_y\right], \tag{S7c}$$

$$\frac{d\langle x_b y\rangle}{dt} = k_b\langle x_b\rangle\langle x_f\rangle\langle y\rangle + k_y\langle B_y\rangle\langle x_b x_f\rangle - \langle x_b y\rangle(k_u + k_b\langle x_f\rangle + \gamma_b + \gamma_y) + k_b\langle x_f y\rangle(N-\langle x_b\rangle). \tag{S7d}$$

Similar to the derivation of the steady state values of first order moments (S5), we solve (S3), (S6) and (S7) at the steady state, and then take the fast binding/unbinding limit to get the expressions the Fano factors for free TF count and target protein count presented in the main text. We use Mathematica [Wolfram Research, Inc., Version 11.3, Champaign, IL (2018).] to solve the second order moments at the steady-state and to take the fast binding/unbinding limit.

## 2. Dynamical equations for two decoy species

In the case of two decoy species, the dynamical equations of the first order moments are given by,

$$\frac{d\langle x_f\rangle}{dt} = \langle B_x\rangle k_x + k_{u1}\langle x_{b1}\rangle + k_{u2}\langle x_{b2}\rangle - k_b\langle x_f\rangle\left[N - \langle x_b\rangle\right] - \gamma_f\langle x_f\rangle, \tag{S8a}$$

$$\frac{d\langle x_{b1}\rangle}{dt} = k_b\langle x_f\rangle(N_1 - \langle x_{b1}\rangle) - \langle x_{b1}\rangle(k_{u1} + \gamma_{b1}), \tag{S8b}$$

$$\frac{d\langle x_{b2}\rangle}{dt} = k_b\langle x_f\rangle(N_2 - \langle x_{b2}\rangle) - \langle x_{b2}\rangle(k_{u2} + \gamma_{b2}), \tag{S8c}$$

where $N = N_1 + N_2$ is the decoy sites, and $x_b = x_{b1} + x_{b2}$ is the total bound complexes. The dynamical equations for the second order moments are

$$\begin{aligned}\frac{d\langle x_f^2\rangle}{dt} =& k_x\langle B_x^2\rangle + k_{u1}\langle x_{b1}\rangle + k_{u2}\langle x_{b2}\rangle + \langle x_f\rangle\left[2k_x\langle B_x\rangle + k_b(N - \langle x_b\rangle)) + \gamma_f\right] - 2k_b\langle x_f\rangle^2\langle x_b\rangle \\ &+ 2\langle x_{b1}x_f\rangle(k_{u1} + k_b\langle x_f\rangle) + 2\langle x_{b2}x_f\rangle(k_{u2} + k_b\langle x_f\rangle) - 2\langle x_f^2\rangle\left[k_b(N - \langle x_b\rangle) + \gamma_f\right],\end{aligned} \tag{S9a}$$

$$\begin{aligned}\frac{d\langle x_{b1}^2\rangle}{dt} =& \langle x_{b1}\rangle\left[k_{u1} + 2k_b\langle x_{b1}\rangle\langle x_f\rangle + \gamma_{b1}\right] + k_b\langle x_f\rangle(N_1 - \langle x_{b1}\rangle) + 2k_b\langle x_{b1}x_f\rangle(N_1 - \langle x_{b1}\rangle) \\ &- 2\langle x_{b1}^2\rangle(k_{u1} + k_b\langle x_f\rangle + \gamma_{b1}),\end{aligned} \tag{S9b}$$

$$\begin{aligned}\frac{d\langle x_{b2}^2\rangle}{dt} =& \langle x_{b2}\rangle\left[k_{u2} + 2k_b\langle x_{b2}\rangle\langle x_f\rangle + \gamma_{b2}\right] + k_b\langle x_f\rangle(N_2 - \langle x_{b2}\rangle) + 2k_b\langle x_{b2}x_f\rangle(N_2 - \langle x_{b2}\rangle) \\ &- 2\langle x_{b2}^2\rangle(k_{u2} + k_b\langle x_f\rangle + \gamma_{b2}),\end{aligned} \tag{S9c}$$

$$\begin{aligned}\frac{d\langle x_{b1}x_{b2}\rangle}{dt} =& 2k_b\langle x_{b1}\rangle\langle x_{b2}\rangle\langle x_f\rangle + k_b\langle x_{b1}x_f\rangle(N_2 - \langle x_{b2}\rangle) + k_b\langle x_{b2}x_f\rangle(N_1 - \langle x_{b1}\rangle) \\ &- \langle x_{b1}x_{b2}\rangle\left[k_{u1} + k_{u2} + 2k_b\langle x_f\rangle + \gamma_{b1} + \gamma_{b2}\right]\end{aligned} \tag{S9d}$$

$$\begin{aligned}\frac{d\langle x_{b1}x_f\rangle}{dt} =& \langle x_{b1}\rangle(-k_{u1} + k_x\langle B_x\rangle) - k_b\langle x_f\rangle\left[(N_1 - \langle x_{b1}\rangle) + \langle x_{b1}\rangle^2 + \langle x_{b1}\rangle\langle x_{b2}\rangle - \langle x_f\rangle\langle x_{b1}\rangle\right] + k_b\langle x_f^2\rangle(N_1 - \langle x_{b1}\rangle) \\ &+ \langle x_{b1}x_{b2}\rangle(k_{u2} + k_b\langle x_f\rangle) - \langle x_{b1}x_f\rangle\left[k_{u1} + k_b(N - \langle x_b\rangle + \langle x_f\rangle) + \gamma_{b1} + \gamma_f\right] + \langle x_{b1}^2\rangle(k_{u1} + k_b\langle x_f\rangle),\end{aligned} \tag{S9e}$$

$$\begin{aligned}\frac{d\langle x_{b2}x_f\rangle}{dt} =& \langle x_{b2}\rangle(-k_{u2} + k_x\langle B_x\rangle) - k_b\langle x_f\rangle\left[(N_1 - \langle x_{b1}\rangle) + \langle x_{b1}\rangle^2 + \langle x_{b2}\rangle\langle x_{b1}\rangle - \langle x_f\rangle\langle x_{b2}\rangle\right] + k_b\langle x_f^2\rangle(N_2 - \langle x_{b2}\rangle) \\ &+ \langle x_{b1}x_{b2}\rangle(k_{u1} + k_b\langle x_f\rangle) - \langle x_{b2}x_f\rangle\left[k_{u1} + k_b(N - \langle x_b\rangle + \langle x_f\rangle) + \gamma_{b2} + \gamma_f\right] + \langle x_{b2}^2\rangle(k_{u2} + k_b\langle x_f\rangle).\end{aligned} \tag{S9f}$$

In the steady state, for a given set of parameters, we solve these equations numerically to obtain the density plot presented in the main text.

## 3. Validity of fast binding/unbinding limit by comparing the analytical and stochastic simulation results

We use kinetic Monte Carlo algorithm due to Gillespie [Gillespie, Daniel T, *Journal of Comput. Phys.* **22**, 403434 (1976)] to solve our stochastic model numerically. For a large dissociation constant, the LNA results for the free TF Fano factor match with the simulation results quite well. However, for a small dissociation constant, a clear deviation is observed near the peak position. The qualitative behavior is the same. Results from stochastic simulations are not that sensitive to the values of the binding rate.

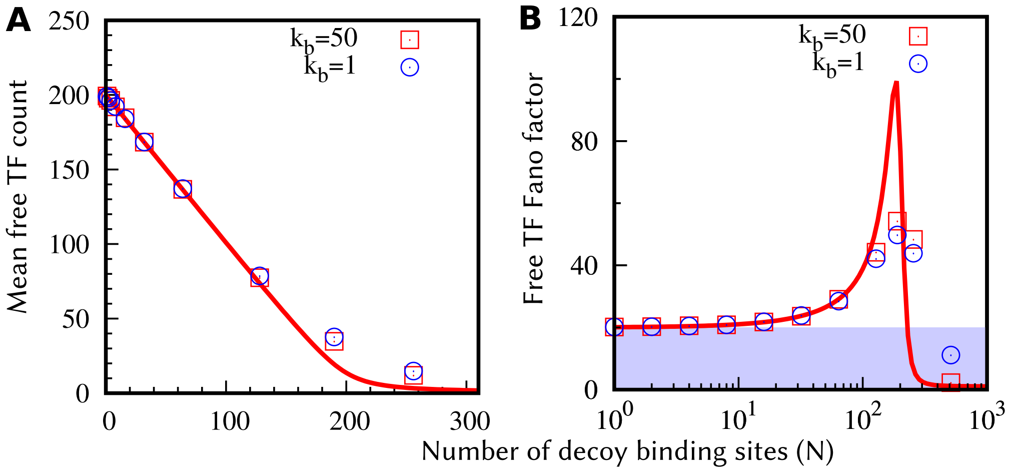


Figure S1: **Effect of fast binding/unbinding limit using stochastic simulation results for free TF counts:** The mean free TF counts (A) and the Fano factor (B) as a function of the total number of decoy binding sites for two values of binding rates $k_b = \gamma_f$ (circular symbols) and $k_b = 50\gamma_f$ (squares). The corresponding lines plotted using the mean (Eq. 6, main text), and Fano factor formula (Eq. 9, main text), obtained using the LNA when $k_b \to \infty$ and $k_u \to \infty$ but $k_u/k_b = k_d$ is finite. Parameters used: $k_x = 10\,hr^{-1}$, $k_d = 1$, $\langle B_x \rangle = 20$, and $\gamma_b = \gamma_f = 1\,hr^{-1}$ per protein molecule.

## 4. The noise in the target protein

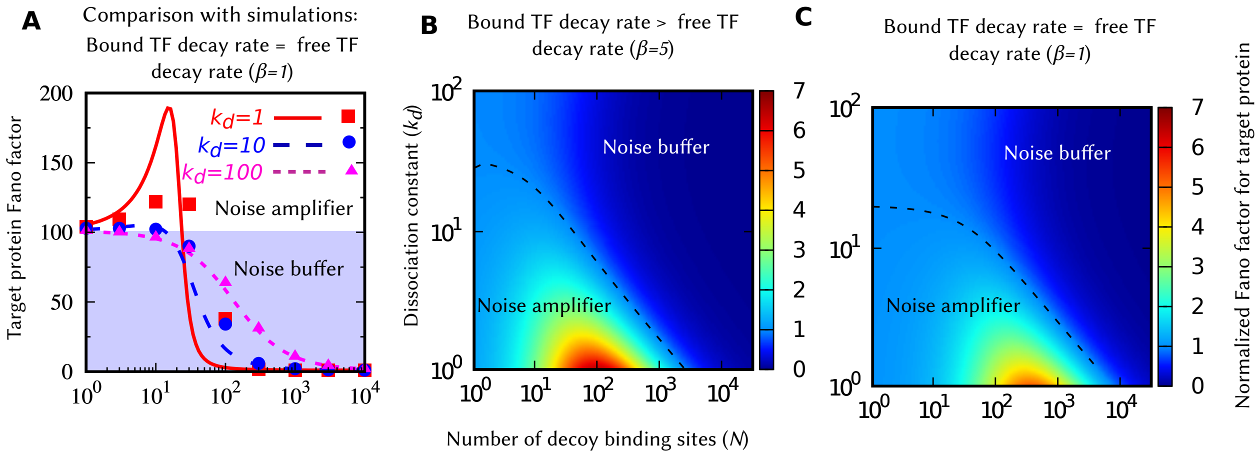


Figure S2: **Noise in the target protein:** (A) Comparison between the analytical results (with lines; Eq. 16, main text) and simulations results (symbols) for $\beta = 1$ for different values of $k_d$. The target protein counts show a noise enhancement similar to free TF count. The analytical results with the linearization approximation predict the noise behavior in target protein correctly for large $k_d$. For a small $k_d$ value, although it qualitatively predicts the noise enhancement, the quantitative match is poor. (B) and(C): The normalized Fano factor ($F_y/F_{y,0}$) for the target protein for constant values $\overline{\langle x_f \rangle}$ and $\overline{\langle y \rangle}$ for $\beta = 5$ and $\beta = 1$, respectively. The magnitude and the region of the noise enhancement are relatively smaller to that of free TF count. Parameters used: $\overline{\langle x_f \rangle} = 20$ and $\overline{\langle y \rangle} = 200$ molecules, $\langle B_x \rangle = 20$, and $\gamma_y = \gamma_f = 1\,hr^{-1}$ per protein molecule. To keep $\overline{\langle x_f \rangle}$ constant, we change $\overline{\langle x_{f,0} \rangle}$ accordingly by varying $k_x$ and obeying Eq. 6, main text. For simulations, $k_b = 50$ per pair of molecules.
